# Paradigm Shifts in Gifted Education: An Examination Vis-à-Vis Its Historical Situatedness and Pedagogical Sensibilities

**DOI:** 10.1177/0016986217722840

**Published:** 2017-07-31

**Authors:** C. Owen Lo, Marion Porath

**Affiliations:** 1 University of British Columbia, Vancouver, British Columbia, Canada

**Keywords:** gifted education, identification, inclusion, development, paradigm, narrative review, history

## Abstract

After nearly a century of development, gifted education has evolved into a complex educational discipline with well thought out pedagogy and research agendas. However, while the number of studies escalates, the field as a whole has been criticized for producing fragmented and piecemeal results. One of the reasons for these shortfalls is that the field has invested little in meta-theoretical aspects, such as historical perspectives and philosophical foundations. This article is a comprehensive review and analysis of the conceptual changes and paradigm shifts in gifted education. Three major paradigm shifts in gifted education were identified—*demystification* (i.e., giftedness as manifested wonders), *identification* (i.e., giftedness as measurable predictions), and *transaction* (i.e., effectuation of human possibilities). Presently, there is still an implicit focus on the identification paradigm despite considerable efforts to shift the focus to creating and sustaining appropriate developmental niches for all individuals. Debates in the field are highlighted to provoke discussion of future directions.

After nearly a century of development, gifted education has evolved into a complex educational discipline with systematic research agendas and well thought out pedagogy (Tannenbaum, 2000). Numerous public and private gifted education organizations have been established (e.g., European Council for High Ability, National Association for Gifted Children/in the United States; Chinese Association for Gifted Education in Taiwan); moreover, there are numerous academic journals^1^ devoted to the study of giftedness and gifted education, and conferences are routinely organized by communities within the field. In the first decade of the 21st century, close to 3,000 studies were indexed in gifted and/or gifted education in the PsycINFO database (Dai, Swanson, & Cheng, 2011). However, while the number of studies in gifted education escalates, many of these studies have been criticized for producing fragmented and piecemeal results (Cohen, 1996; Renzulli, 2012). One of the reasons for this fragmentation could be that the field, as a whole, has invested little in meta-theoretical aspects such as historical perspectives and philosophical foundations (Cohen, 1996, 2006). As Cohen, Ambrose, and Powell (2000) cautioned, “Without a solid conceptual base and theoretical awareness, researchers and practitioners tend to develop conceptual blind spots and ignore important aspects of giftedness and talent” (p. 331). Emerging as a trend in recent years, some scholars are making efforts to generate meta-theoretical discourses and encourage the community in dialogue on topics such as worldview, ideology, and paradigm shifts (e.g., Ambrose, 2012; Heller & Schofield, 2000; Mönks, Heller, & Passow, 2000). For example, Dai and Chen (2013, 2014) rendered an analytical account of paradigm shifts in gifted education, providing an in-depth analysis of the paradigmatic differences of three major gifted education approaches (i.e., gifted child approach, talent development approach, and differentiation approach). They made explicit the underlying assumptions and goals of each approach and provided deep implications for both research and practice.

Since Kuhn’s (1962/1996) landmark explanation of how paradigm shifts constitute the structure of scientific revolution, the term “paradigm” is commonly mentioned in research articles (Göktürk, 2015). In Kuhn’s view, a paradigm shift involved radical changes in how a field conceptualizes itself—changes that were resisted by the established community. Capra (1996) broadened Kuhn’s notion and defined paradigm as “a constellation of concepts, values, perceptions, and practices shared by a community, which forms a particular vision of reality that is the basis of the way the community organizes itself” (p. 6). In other words, a paradigm is the “broadest unit of consensus within a science and serves to differentiate one scientific community (or sub-community) from another. It subsumes, defines and interrelates the exemplars, theories, and methods and tools that exist within it” (Ritzer, 1975, p. 9). Therefore, an investigative research paradigm importantly functions as tinted glass through which a researcher perceives, questions, and interprets events. This article’s objective is to go beyond a paradigmatic comparison of approaches with gifted education; it provides a meta-theoretical account of paradigm shifts in conceptual understandings of giftedness and gifted education that can inform research and practice. While this study presents a narrative review (Collins & Fauser, 2005; K. Jones, 2004), the process in which the theoretical framework emerged was akin to the generation of a grounded theory (Glaser, 2002; Glaser & Strauss, 1967; Holton & Walsh, 2017; Lo, 2014a, 2016), a heuristic process dependent on *constant comparisons* and the researcher’s *theoretical sensitivity* (i.e., field knowledge and capability to synthesize/theorize, see Lo, 2014a, 2016). Crucially, these comparisons and sensitivity served as the catalyzing tool for searching and integrating literature in the process of generating themes and seeking theoretical saturation (Glaser & Strauss, 1967; Holton & Walsh, 2017; Lo, 2016).

In this heuristic theorizing process, we started with a preliminary paradigm shifts framework outlined in an article published by the first author (Lo, 2014b) and used this preliminary understanding as a touchstone for searching and analyzing the literature identified in this first iteration. In this iteration, we included the scholarly work from (a) key contributors to gifted curricula and pedagogies and (b) thinkers whose work constitutes important sources of historical knowledge and conceptual changes in the field. In particular, we expanded our search to include contemporary work on inclusive schooling vis-à-vis gifted education. The body of literature reviewed in this iteration consisted of purposeful database keyword searches (using *Academic Search Complete*) and our collective field knowledge. The second-round literature review started when we made connections between epistemology (i.e., knowing) and pedagogy (i.e., learning and teaching). In this iteration of analysis, we included general developments in both academic research and public education and focused on examining the nexuses among these various (yet connected) lines of development to provide a broader perspective that went beyond and above gifted education. After two iterations of analysis, we registered three epistemological strands as the points of departure for comparison and for constructing an account of the historical emergence of gifted education (that is, how mainstream academic and educational movements intersect with shifting concepts of giftedness and influence the development of gifted education).

The paradigm shifts framework of gifted education (henceforth PSF-GT) we present in this article consist of three broad stages in how the concept of giftedness is understood and studied—namely, *demystification, identification*, and *transaction*—that are epistemologically distinct from each other. Importantly, the vocabulary of the paradigms was chosen to indicate the embedded teleologies (i.e., purposes for research and/or practices) and an action-oriented ideology. In the following discussion, we elucidate each paradigm vis-à-vis its historical situatedness (e.g., educational and academic movements) and pedagogical sensibilities. To further understanding, we begin with a precursory introduction of PSF-GT (see Table 1) before proceeding to a detailed account of explanation. A few decades before, the modern onset of gifted education in the 1920s, giftedness was perceived as a scientific topic under the then dominant influence of positivism. Before giftedness was studied in a systematic way, it was considered as mystical divinity and was often associated with superstition and neuroses. Early studies of giftedness began to demystify the construct; it was considered examinable and therefore could be unpacked and discovered through systematic investigations. During this early context, studies on giftedness were predominantly confined to the pursuit of scientific discovery. However, this scientific enthusiasm did not find a strong foothold in education because the concurrent movement to standardize education (see B. Davis, Sumara, & Luce-Kapler, 2015) was concerned more about mass-producing a quality work force than fulfilling individual needs. In the 1920s, the emergence of intelligence tests, in combination with an education movement that urged authenticity in recognizing individual differences via educational placement (see B. Davis et al., 2015), set the foundation of modern gifted education. In the 21st century, while identification remains centrally prominent in gifted education and reflects an overarching postpositivist influence, the notion of who may be identified as gifted has been broadened by a confluence of modes of critical inquiry (e.g., epistemological constructivism, social constructionism, critical theory [cf. Cohen, 2006; Cohen et al., 2000]), and a democratic citizenship education movement (see B. Davis et al., 2015) that aims to remove undemocratic constraints (e.g., gender, race, socioeconomic status) and empower individuals who are considered disadvantaged. Furthermore, an emergent alternative interpretation of giftedness that is distinct from an identification-based ideology is drawing attention from scholars. Rather than focusing on identifying students who are gifted, this new interpretation sees giftedness in terms of functional transactions between an individual and his or her environment. In other words, giftedness becomes a pedagogical goal achievable by all rather than measurable predictions for some. This transaction-based paradigm (e.g., Barab & Plucker, 2002; Eyre, 2011; Hymer, 2009, 2012; Hymer, Whitehead, & Huxtable, 2009; Lupart, 2012) conceptualizes giftedness as functional conditionality (that should be locally realized) rather than possessed conditions (to be identified). This new wave of thinking corresponds to systemism^2^ (see Bunge, 1996, 2000; Capra & Luisi, 2014; Choi, 2011; B. Davis et al., 2015; Pickel, 2011; von Bertalanffy, 1968; Wan, 2011) that abandons the reductionist worldview (i.e., seeing the world in mechanical components) and discerns the (dys)functional relation between environment, agent, and embedded sociocultural structures.

**Table 1.** Paradigm Shifts in Gifted Education (PSF-GT).

	Action-oriented ideology	Epistemological framework	Conceptual undertaking	Preferred terminology
Early context	Demystification	Positivism	Heredity	Genius
				Prodigious
Modern onset	Identification	Postpositivism	Measurability	Gifted
Current shifts	Transaction	Systemism	Conditionality	Advanced
				More able

## Early Context: The Emergence of a Demystification Paradigm

Historically, people who possess outstanding abilities have fascinated society. As one can easily imagine, a fledgling hunter or a talented cave painter drew much admiration from his or her tribesmen in a prehistoric society (Stanley, 1976). In Greece, Plato described men with superior intellect as men of gold, distinct from those of silver, iron, or brass. In Plato’s ideal world, these golden children were offered a program that required mastery of science, philosophy, and metaphysics to increase their capacity for leadership (Freeman, 1979; Tannenbaum, 2000). In China, starting in the Tang Dynasty (AD 618), child prodigies were sent to a conservatory set by the imperial court where their talents and giftedness could be elevated (Tsuin-Chen, 1961). Dating back to 500 BC, Confucius, the most famous pedagogue in China, proposed that education should be available to all children with programs tailored to individual differences. However, neither Plato’s nor Confucius’s ideas were publicly mandated and implemented. Educational resources in ancient times were available only to the upper class (G. A. Davis, Rimm, & Siegle, 2011; Tannenbaum, 2000).

It is clear that eminence and exceptional achievements have always evoked interest; documentation is found in folklore, art, theology, philosophy, and, more recently, natural, and social sciences (Jolly & Bruno, 2010; Ziegler & Heller, 2000). Influenced by British empiricist David Hume^3^ (1711-1776), Auguste Comte^4^ (1789-1857) put forth his influential law of three stages (Comte, 2009) in an effort to depict the intellectual progress of human beings. In the first stage—the theological (or fictitious) stage—humankind understood the world largely through superstition and/or animism. Evolving into the second stage—the metaphysical (or abstract) stage—humankind extended their explanatory modes by appealing to abstract entities or forces (such as momentum). Comte’s final stage is a positive (or scientific) stage. In this stage, scientific thinking was introduced; humankind generated knowledge based on facts derived from observation and experience. The belief was that there were natural laws to which all phenomena were subject. Comte’s broad stages seem to apply to the ways in which giftedness has been imagined and reasoned about in our history. In ancient times, giftedness and talents were interpreted through religious lenses^5^ and explained via divinity and mythology (Freeman, 1979; Grinder, 1985; Jolly & Bruno, 2010; Robinson & Clinkenbeard, 2008). Ancient Western cultures, considered talented individuals as people touched by divinity and believed that Muses divinely inspired exceptional work (Robinson & Clinkenbeard, 2008). Gradually, giftedness and talents came to be understood in more abstract terms, such as personal aptitude, extrasensory causes, or excessive neuroses (Grinder, 1985; Passow, Mönks, & Heller, 1993; White, 2000; Ziegler & Heller, 2000). As a case in point, Lombroso (1891) associated geniuses with neuroses that often resulted in pathological manifestations. As the Scientific Revolution ushered in a zeitgeist of positive philosophy and empiricism (J. Henry, 2004), systematic thinking and quantification of variables believed to be associated with intelligence became the framework for understanding giftedness and talents (Grinder, 1985). A paradigm of demystification of giftedness emerged, in which scientists and scholars strived to unpack individual differences through systematic investigation and measurement.

The emergence of the demystification paradigm can be traced back to Victorian scientists’ work on biological heredity, genetics, and species (Tannenbaum, 1958), for example, Charles Darwin’s (1809-1882) work on the origin and evolution of species and Gregor Mendel’s (1822-1884) work on laws of inheritance (see Harris, 1923). Along the same lines, Francis Galton (1822-1911) researched the implications of Darwin’s theory and assumed a biological and genetic etiology of giftedness. He hypothesized that eminent achievements resulted from one’s intellectual heritage. To test his hypothesis, Galton devised several methods and apparatuses designed to capture the keenness of one’s sensory and functional abilities (e.g., vision, audition, olfaction, tactility, and reaction time). He concluded that giftedness was largely inherited (Galton, 1869). While Galton’s work is considered distasteful today due to the strong connotations of racism, classism, and eugenics, his studies on giftedness did challenge some customary attitudes and mythical thinking about giftedness (e.g., that geniuses were physically unsound and mentally distressed). The systematic anthropometric and biographical methods Galton adopted for investigating giftedness resulted in him being credited as the founding “grandfather” of 20th-century gifted education (Goldstein, 2012; Guilford, 1936; Jensen, 2002; L. V. Jones & Thissen, 2006; Stanley, 1976).

The momentum of quantifying individual differences continued in the late 19th and early 20th centuries. While still focused on the goal of understanding and measuring individual differences in intelligence, Alfred Binet (1857-1911) viewed intelligence differently (Siegler, 1992). Unlike his contemporaries, such as Galton (1869, 1874) and Cattell (1890, 1903), who predominantly focused on easy-to-measure sensory and functional abilities, Binet emphasized complex mental processes (e.g., reasoning and comprehension) and treated these mental processes as the main locus of individual differences in intelligence (Robins, 2010; Siegler, 1992; White, 2000; Wolf, 1973). He was appointed by the French government to develop an intelligence test that could be used to identify students who lagged behind benchmarks for learning (Siegler, 1992). In collaboration with Theophile Simon, Binet created the Binet–Simon Scale (1904) and introduced the idea of mental age to define a child’s intellectual progress in relation to his or her peers (Fancher, 1985; L. V. Jones & Thissen, 2006; White, 2000). Although Binet’s tests were not intended for gifted children, his contribution was considered epochal because the tests introduced new language for the construct of intelligence and its measurement (Hollingworth, 1936). For this, Binet is commonly recognized as the father of the intelligence test (Siegler, 1992).

Whereas research focused on, or associated with, giftedness was evident in the late 19th and early 20th centuries (e.g., Cattell, 1903; Galton, 1869, 1874), educational implications were not. The notion of giftedness was confined to the study of individual differences; pedagogical relevance was not considered. For example, although the word “gifted” was adopted in Galton’s (1869) book *Hereditary Genius*, it was used to indicate adults who possessed endowed gifts for achieving eminence. Parallel to the emergence of the demystification paradigm, in the Western world a standardized education movement driven by the Industrial Revolution occurred (B. Davis et al., 2015; Dewald, 2004); the purpose of education was to provide the immense labor force needed by the increasing number of modernized factories. Conceivably, catering to individual differences could not be a chief goal of education when the operation addressed largely standardized outputs and capacity for mass production.

In sum, the core undertaking of the field in the demystification paradigm was to use scientific methods to unpack the nature of human intelligence. As the research progressed, understanding of giftedness and talents started to grow away from misbeliefs and superstitions.

## Modern Onset: The Emergence of the Identification Paradigm

The advent of a quantifiable measuring device for identifying gifted children was crucial to the continuation and refinement of gifted education programs. (R. G. Miller, 2009, p. 369)

The confluence of psychometric studies on individual differences and a compulsory education movement created momentum for formal gifted education (Borland, 2005; L. J. Coleman, Sanders, & Cross, 1997). With strong roots in empiricism, the gold standard for answering educational research questions became psychometric methods. By the 1920s, assessment of individual differences had become “a well-established means by which educational psychologists could help shape school practices and educational policy” (Lagemann, 2000, p. 93). Hollingworth (1938) noted the following:

The greatest contribution made by psychology to education since the turn of the century has been this power to measure minds of greater caliber. . . . The idea of finding the gifted in childhood is therefore not new. It is the power to do so that is new, and that brings to educators the new opportunities and responsibilities which we have been discussing. (p. 306)

Here, Hollingworth (1938) pinpointed the applicability of intelligence tests to pedagogy for students who could be defined as gifted by these measures and benefit from advanced curricula. Concurrently, the standardization of public education neared completion. With the presence of an expanded middle-class, public education progressively became compulsory (B. Davis et al., 2015). As school enrollments increased, diversified student bodies and an escalating multitude of educational needs followed naturally. Educators and researchers started to pay attention to students whose learning needs and speed exceeded a uniform, age-related curriculum (Robins, 2010). Sophisticated psychometric measures influenced the emergence of modern gifted education, partially in response to the pedagogical mission to differentiate curriculum for a range of ability levels (B. Davis et al., 2015; Goldstein, 2012; Robins, 2010). For example, a preparatory school designed specifically for gifted students in Worcester, Massachusetts was documented as early as 1901 (see G. A. Davis et al., 2011). In 1911, the U.S. Bureau of Education reported that about 6% of cities had some form of special programs for gifted children (see Nazzaro, 1977). By 1920, approximately two thirds of larger cities in the United States had created some type of program for gifted students (Colangelo & Davis, 2003). Also in the 1920s, the term “gifted” started to appear on the title pages of educational books (e.g., T. S. Henry, 1920; Hollingworth, 1926; Stedman, 1924). These early seeds of gifted education provisions in tandem with the systematic investigation of gifted individuals led by scholars such as Lewis Terman (1877-1956) and Leta Hollingworth (1886-1939) set in motion the work of the field of gifted education.

Following the torch lit by Francis Galton, Terman was passionate about studying gifted students. Combining Alfred Binet and Theodore Simon’s (1904) construct of intelligence as complex reasoning ability and their method of testing intelligence with William Stern’s (1914) concept of intelligence quotient,^6^ Terman published revisions of the Binet–Simon Scale (see Terman, 1916; Terman & Merrill, 1937) that quickly changed the practice of education (Jolly, 2008; Stanley, 1976). That is, educators were able to make selections of students based on their performance on intelligence tests and design programs that better addressed the learning needs of students that could not normally be met by a regular curriculum. For many decades, Terman’s intelligence tests were “the standard for ascertaining the mental age and IQ of persons” (Stanley, 1976, p. 39). Terman also began a series of longitudinal studies in the 1920s, following his sample of 1,500 children who had an average IQ of 151 (Kaufman, 2013) over decades. At midlife, the “Termites” (Kaufman, 2013) had impressive educational and career achievements and were healthy, socially well adjusted, and very satisfied with life (Feldman, 1984; Kaufman, 2013). Terman’s systematic investigation became “the most comprehensive compilation of empirically gathered data on gifted education of its time” (Jolly, 2008, p. 28). Essentially, Terman’s longitudinal studies can be considered a paradigmatic shift. His studies set out to dispel the lingering myths and misbeliefs surrounding gifted children and succeeded in putting to rest the images of physically weak and socially inept gifted individuals (White, 2000). That said, his longitudinal studies have also been criticized as elitist and eugenic (Stoskopf, 2002). Contemporary scholars have noted that Terman’s sample was predominantly White and middle-class, which could have accounted for their impressive achievements in later life (e.g., Leslie, 2000; Vialle, 1994). The sample also was nominated by teachers who favored high achieving well-behaved students, and excluded creative thinkers like Luis Alvarez and William Shockley who went on to win Nobel prizes (Kaufman, 2013). No such distinction accrued to participants in Terman’s study. In historical context, however, Terman’s belief that gifted children could be identified through intelligence tests had significant implications for education and gave gifted education a foothold in academia (Eyre, 2011; Jolly, 2008; Subotnik, Olszewski-Kubilius, & Worrell, 2011; Vialle, 1994). The work by Terman, Hollingworth, and their contemporaries drew attention to the needs of children who are developmentally advanced (Keating, 1991), attention that was largely based in the identification-based ideology that dominated gifted education in the past century. However, practices of identification have undergone several conceptual changes (Lo, 2014b), described subsequently.

### From Simplicity to Complexity

At the beginning of modern gifted education, giftedness was judged almost solely by IQ and consisted of a very small fraction of the student body. For example, Terman (1925) and Stedman (1924) decided that individuals with an IQ ≥140 should be deemed gifted; Hollingworth (1926) favored 130, whereas Whipple (1919) chose 115. Terman (as cited in Hollingworth, 1926) also put forth an IQ-based stratification that depicted the educability of students, from “feebleminded” (below 70 IQ) to “genius or near genius” (above 140 IQ).

Not long after, scholars started to reflect on the limitations of an IQ-based construct of giftedness. As early as the 1920s, Hollingworth (1926) noted the possibilities of giftedness in various domains and argued that students “may be far more excellent in some capacities than others” (p. 202). Later, Bentley (1937) called for advanced curriculum for students who demonstrate aptitude in specific areas such as art, music, or mathematics. More formally, Witty (1958) stated a definition that included general intellectual abilities as well as specific talents (e.g., arts, writing, and leadership) in an annual yearbook of the National Society for the Study of Education in the United States. Moreover, creativity—a dimension commonly included as part of the definition of giftedness today (e.g., Mönks, 1992; Renzulli, 1978; Sternberg, 1996, 2005)—started to receive attention in the field of gifted education after Guilford made an APA presidential address on creativity in 1950. As a case in point, in 1972, when the United States announced its first federal definition, giftedness was defined as:

Children capable of high performance include those with demonstrated achievement and/or potential ability in any of the following areas, singly or in combination:

1. General intellectual ability
2. Specific academic aptitude
3. Creative or productive thinking
4. Leadership ability
5. Visual and performing arts
6. Psychomotor ability. (Marland, 1972, p. 2)

This conceptual wave of seeing intelligence in complex multidimensional terms hit its climax when Gardner (1983) proposed a theory of multiple intelligences that included verbal–linguistic, logical–mathematical, spatial, musical, bodily kinesthetic, interpersonal, and intrapersonal competencies. Gardner (2006) later added naturalistic and existential intelligences, for a total of nine. Each type of intelligence reflects a competency thought to have its own developmental trajectory and unique neural architecture; each also reflects the values of and support provided by different cultures. Gardner criticized the ways in which intelligence had been singularly conceptualized (e.g., Piaget’s [1970] view of intelligence as general operational schema that underpin all thought) and addressed in school (e.g., predominant emphasis on linguistic and logical–mathematical aspects of symbol use).

Contemporary views of giftedness posit that it is dynamic and socially constructed (Borland, 1997, 2013; Callahan & Hertberg-Davis, 2013, Lo, 2014b; Matthews & Foster, 2006; E. M. Miller, 2013; Sutherland, 2012; VanTassel-Baska, 2005; Ziegler, Stoeger, & Vialle, 2012). Newly emerged theories of intelligence(s) by and large reflect this conceptual trend. For instance, from a pragmatist view and focusing on cognitive functioning, Sternberg (1985, 1999) posited three types of intelligence, namely analytic, synthetic, and practical, which he viewed as functionally different from each other. The cognitive processes underlying each intelligence were hypothesized to account for their functional differences. Of note, despite the common awareness of the dynamic and socially constructed nature of intelligences (e.g., Gagné, 1995; Gardner, 1983; Mönks & Katzko, 2005), disagreements on the definitions of intelligence still prevail in the field. To some, intelligence is still conceptualized as an overarching general ability (g) that is biologically based and predicts success in life (e.g., Carroll, 1993; Gottfredson, 1998, 2002; Jensen, 1998).

In sum, the early IQ-based definition of giftedness was certainly a product of its time. It was considered the scientific way under the influence of positivism (Borland, 2005; L. J. Coleman et al., 1997) in which giftedness was viewed as a natural phenomenon measurable by means of objective tests and rating scales. The evolving complexity and iterations of the definition that we are aware of today, however, reflect an ongoing dialectical understanding of a complex social construct (cf. symbolic interactionism [Blumer, 1969] and social constructionism [Burr, 2003; Crotty, 1998/2011]). In other words, the field as a whole is engaging in a collective meaning- and sense-making process of what gifted education can and should be (Borland, 2005; Cramond, 2004; Lo, 2014b).

### From Being to Becoming

“Nature gives no gifts. . . . Genetic potentials unfold in interaction with stimulating experiences structured by parents, family, home, schools, teachers, and curricula” (Feldhusen, 2005, p. 64). Increasingly, research and practice are moving from a static and absolute notion of who is gifted—or not—to recognition of the importance of the interactions between individuals and the environment that contribute to the competency. There is acknowledgement of the intricate nuances of human development as it unfolds over the life span (Horowitz, Subotnik, & Matthews, 2009) and increased understanding of the complexity of the developmental scaffolding needed for children to develop optimally (Keating, 2009; Shonkoff & Phillips, 2000). Evidently, there is a change of focus from “being” gifted to “becoming” gifted (Lo, 2014b, p. 286), largely influenced by developmental psychology and epistemological constructivism (see Ültanir, 2012).

In 1930, Vygotsky proposed an important concept, the zone of proximal development, the zone that represents the difference between what children can accomplish independently and what they can accomplish with sensitive support from an adult or more competent peer. In Vygotsky’s view, intelligence is subject to change and is not readily observable without proper scaffolding. Similarly, Fischer and Pipp (1984) noted that children will not demonstrate their optimal level of understanding without sensitive support, a principle that is foundational to Vygotskian-influenced dynamic assessment. Dynamic assessment is not widely practiced in gifted education. However, it does have the potential to inform educators about the nature of scaffolding (cues and types of support) needed by children to achieve optimally. It is also an appropriate strategy to use in supporting the development of the many abilities that relate to human accomplishment and has the potential to contribute significantly to our knowledge of how these abilities are encouraged and sustained across the life span (Dweck, 2009).

Epistemological constructivism brought rigor to our understanding of the processes of “becoming.” Studying the “what” and “how” of children’s thinking—the nature of thinking in different domains, how that thinking is consolidated, integrated, and applied, and how it develops in complexity over time—has led to better understanding of the conceptual underpinnings of thought and the influences that support different developmental trajectories (e.g., McKeough, Genereux, & Jeary, 2006; Okamoto, Curtis, Jabagchourian, & Weckbacher, 2006; Porath, 2006a, 2009). This perspective captures the emergent nature of giftedness (Porath, 2006b)—an understanding central to moving us more firmly into a focus on “becoming.”

Giftedness is currently viewed as “dynamic, contextual, and emergent” (Dai, 2010, p. 21), a view that owes much to developmental psychology, through its study of the startling developmental diversity among children with high IQs (Horowitz, 2009; Lubinski & Benbow, 2000; Matthews, 1997), systematic investigation of other intelligences (e.g., Case, 1992; Liben, 2009; Porath, 1996, 1997, 2003; Winner, 2009), and the roles of complexity in the environment (Barab & Plucker, 2002; Hymer, 2009; Jackson, 2000) and social affordances (Keating, 2009) in supporting optimal development of giftedness and talents. The definition of giftedness has been extended from a narrow perspective (i.e., seeing giftedness as a stable and unchangeable trait) to a more expansive perspective in which giftedness is viewed as taking multiple forms and developing over time (Morelock, 1996). Theoretical models that address potentiality and developmental issues abound in the gifted education literature (e.g., Feldhusen, 1994, 1998, 2005; Gagné, 1995, 2003, 2004; Renzulli, 1978, 1986, 2005; Tannenbaum, 2003). While these models address the importance of developmental issues concerning giftedness, they commonly reflect an identification-based ideology. For example, Gagné (1995, 2003, 2004) proposed the Differentiated Model of Giftedness and Talent that highlights the importance of the amalgamation of natural abilities, intrapersonal catalysts, developmental process, environmental catalysts, and change in a gifted individual’s optimal development. Meanwhile, he also put forth “a precise threshold of 10% prevalence to separate those who should be labeled from those who should not” (Porath, 2004, p. 154), further categorizing those who should be labeled into various levels from mild to extreme. Some contemporary views on human development question these arbitrary categorizations and are in favor of creating and sustaining appropriate developmental niches for all individuals (e.g., Horowitz, 2000), an aspect discussed later in this article (cf. transaction paradigm).

### From Exclusivity to Diversity

A more recent conceptual wave that has occurred in the identification paradigm is aligned with critical theory perspectives in the social sciences and the democratic citizenship education movement (Ambrose, 2012; B. Davis et al., 2015; Fay, 1987; Lo, 2014b). This conceptual wave speaks to the inclusion and empowerment of gifted students who are considered disadvantaged and the engagement of researchers and educators in analysis of the injustice and undemocratic constraints implicitly and explicitly embedded in our system. From the outset, gifted education has been criticized as elitist and a contributor to social inequity (Matthews & Dai, 2014; Oakes, 2005; Sapon-Shevin, 2000; Sutherland & Stack, 2014). It is not surprising that the public finds gifted education distasteful if we look into Galton’s (1869) and Terman’s (1925) eugenic research agendas and the later scholarly output that followed their work closely, such as the work of Cravens (1988, 1991), Minton (1988), and Hunt (1961). From a social justice perspective, education should provide access to not only knowledge but also opportunities for students (B. Davis et al., 2015). This conceptual wave encourages critical consciousness toward society and envisions new possibilities through empowering gifted individuals who are oppressed in our current society and considered disadvantaged due to structural injustice and historical misunderstanding (Cohen et al., 2000; Lo, 2014b). In light of this conceptual wave, many field scholars engaged in studying and provoking conversations on issues surrounding diversity and social justice in education, such as gender and sexual orientations (e.g., Hutcheson & Tieso, 2014; Maurer, 2011; Reis, 1987, 1995; Sedillo, 2013); cultural, ethnic, and linguistic backgrounds (e.g., Ford, 2004, 2005; Ford, Grantham, & Whiting, 2008; Omdal, Rude, Betts, & Toy, 2011; Worrell, 2014; Worrell & Dixson, 2016); demographics and geography (e.g., Floyd, McGinnis, & Grantham, 2011; Howley, Howley, & Pendarvis, 2003); poverty (e.g., Burney, & Beike, 2008; Kitano, 2003; R. G. Miller & Gentry, 2010; Peters & Gentry, 2010; Warwick & Matthews, 2008); and dual exceptionalities (e.g., Assouline & Whiteman, 2011; Callard-Szulgit, 2008; Dole, 2001; Foley-Nicpon, Assouline, & Stinson, 2012; Lo, 2014b; McCallum et al., 2013; Nielsen, 2002). Currently, it is commonly believed that educators in gifted education should be mindful of existing and potential constraints posed by our society. Consequently, this conceptual wave has broadened the notion and identification of giftedness.

In 1993, the U.S. Department of Education revised their definition of giftedness and noted the importance of recognizing gifted pupils from culturally and socioeconomically disadvantaged groups and communities.

Children and youth with outstanding talent perform or show the potential for performing at remarkably high levels of accomplishment when compared with others of their age, experience, or environment. These children and youth exhibit high performance capacity in intellectual, creative, and/or artistic areas, and unusual leadership capacity, or excel in specific academic fields. They require services or activities not ordinarily provided by the schools. *Outstanding talents are present in children and youth from all cultural groups, across all economic strata, and in all areas of human endeavor.* (p. 19, italics added)

In British Columbia, Canada, the definition of giftedness further included students who exhibit dual exceptionality.

A student is considered gifted when she/he possesses demonstrated or potential abilities that give evidence of exceptionally high capability with respect to intellect, creativity, or the skills associated with specific disciplines. Students who are gifted often demonstrate outstanding abilities in more than one area. They may demonstrate extraordinary intensity of focus in their particular areas of talent or interest. *However, they may also have accompanying disabilities* and should not be expected to have strengths in all areas of intellectual functioning” (British Columbia Ministry of Education Special Education Services, 2006, p. 53, italics added).

To accommodate the notion of broadened inclusion, new approaches have been proposed to help identify gifted students who may not have been recognized by more traditional approaches due to social constraints and/or disadvantage. For example, Wiley and Brunner (2014) suggested using nonverbal intelligence tests to overcome the verbal development problems associated with cultural backgrounds and socioeconomic status, such as the Test of Nonverbal Intelligence (L. Brown, Sherbenou, & Johnsen, 1997), Universal Nonverbal Intelligence Test (Bracken & McCallum, 1998, 2016), Naglieri Nonverbal Ability Test (Naglieri, 2003), and Cognitive Abilities Test—Nonverbal subset (Lohman & Hagen, 2001). Gottfredson (2004) and Lohman (2005) both adopted dynamic and flexible definitions and proposed multimodal approaches to address underrepresentation of minorities in gifted identification.

### Beyond IQ: Section Summary

Before we can educate the genius, we must discover him in childhood. (Hollingworth, 1938, p. 306)

**Figure 1.**
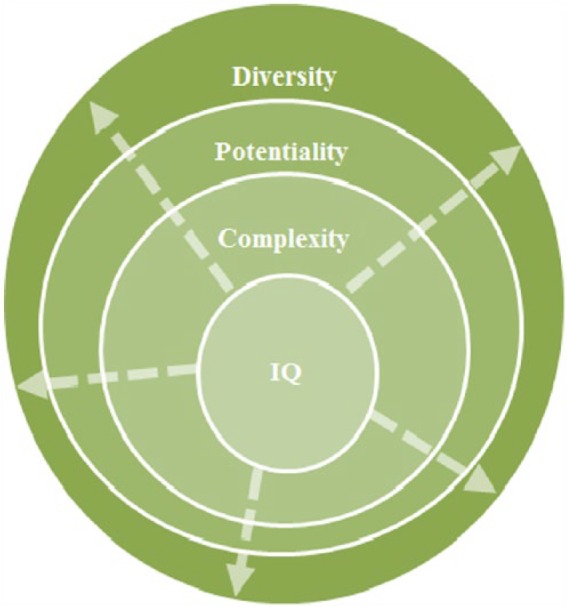
A broadened notion of giftedness.

Simply and fittingly, Hollingworth’s words summarize the core thesis of the identification paradigm. Commencing in the 1920s, intelligence tests started an identification-based ideology and enabled the beginning of formal gifted education. While some of the early foci were criticized for promoting elitism and centering on a fixed view of ability (Borland, 1997; Callahan & Hertberg-Davis, 2013; Feldman, 1979, 2003; Sapon-Shevin, 2000; Sutherland & Stack, 2014), conceptual waves that occurred in the past few decades broadened the notion of giftedness (see Figure 1).

While the identification-based ideology reflects deep-rooted postpositivism (i.e., there are gifted students who await proper identification), the ways in which giftedness is defined have been influenced by some contemporary modes of critical inquiry, such as social constructionism (e.g., Burr, 2003), symbolic interactionism (e.g., Blumer, 1969), social constructivism (e.g., Vygotsky, 1930), and critical theories (e.g., Fay, 1987). Consequently, current identification practices include a broader range of students. Of note, this broadened inclusion is different from “inclusive education,” which is discussed in the next paradigm shift.

Today, with greater consideration of diversity and democratization in understanding giftedness, most educators and researchers in the field see giftedness as much more than an IQ cutoff (Borland, 1996; McGlonn-Nelson, 2005; Morelock, 1996; Sternberg, 1985). Corresponding to this broadened notion of giftedness, a teacher’s role has undergone some changes, moving from a more passive role (e.g., a spectator searching for various forms of giftedness and talents) to a more constructive role (e.g., a scaffolder who make efforts to ensure the “becoming” process of giftedness and talents), to an active emancipating role (e.g., an empowerer who intends to amend injustice and lift undemocratic constraints that might have oppressed gifted students considered disadvantaged). While an identification-based ideology is still prevalent in current gifted education practices, an ecology- and system-based paradigm that speaks to an ideology of cultivating gifts rather than identifying gifts has emerged in recent years (Barab & Plucker, 2002; Eyre, 2011; Hymer, 2009; Hymer et al., 2009; Renzulli, 2010; Sutherland, 2012).

## Current Shifts: The Emergence of the Transaction Paradigm

As gifted education becomes more concerned about appropriate programs and services that can bolster achievement in schools for both gifted and other populations and less concerned about precise identification of who is gifted, the emphasis turns then to what works—what programs and services are likely to produce the greatest learning for students? (VanTassel-Baska & Wood, 2010, p. 345)

VanTassel-Baska and Wood’s notion of what the primary focus of contemporary gifted education should be highlights of a growing interest in making education gifted, that is, in providing an education that allows all students to have rich and varied educational experiences matched to their interests and abilities, and a growing distaste for identifying a special population who would traditionally be served in a gifted program. In recent years, more researchers and educators have started to reflect critically on the underlying assumptions of an identification-based ontology of giftedness and the practices that follow. First, the strong focus on identification that reflects a reductionist belief that giftedness is something that a child either possesses or not has been challenged (Borland, 2005, 2009; Eyre, 2011; Hymer, 2009; Lupart, 2012; Lupart & Webber, 2012; Matthews & Dai, 2014; Plucker & Barab, 2005, Porath, 2012); identification-based practices reflect an understanding of giftedness as an either/or trait, rather than a dynamic state based on learning needs in relation to the environment in which a student is situated. In other words, giftedness has been considered as a fixed normative *condition* (that awaits proper identification) rather than fluid *conditionality* that speaks to the relational learning needs that arise from mismatches between a learner and a learning environment. Second, the extent to which the gifted label helps in facilitating communication between research and practice has also been questioned (Borland, 2009; Eyre, 2011; Lo, 2014b; Peters & Matthews, 2016; Sutherland, 2012) given its educationally nondescript nature (Peters, Kaufman, Matthews, McBee, & McCoach, 2014). Moreover, and perhaps more important to some, the tenability of identification-based practices has also been challenged on axiological grounds. For instance, the question of whether the label implies elitism and entrenches social inequality has been raised, as has the question of whether the field is progressing in the ways in which our societies and academic communities are evolving. “This simplistic dichotomization of humanity into two distinct, mutually exclusive groups, the gifted and the rest (the average? the non-gifted? the ungifted?), is so contradictory to our experience of life” (Borland, 2013, p. 75). Eyre (2011) also argued that while the label can sometimes be perceived as confirmatory and enabling, the very existence of the label also has, even though unintentionally, detrimental effects on those judged as “not-gifted” (such as a lowering of confidence). Similarly, Jackson (2000) raised the question of whether we should direct our efforts to addressing categorization, efforts that have not moved the field forward, or to designing rich environments with emphases on social justice and social equality that benefit all children. In fact, many scholars have argued that the gifted label should be attached to programs rather than individuals (e.g., Borland, 2013; Matthews & Dai, 2014; Olszewski-Kubilius & Calvert, 2016; Peters et al., 2014).

Reflecting on the limitations and constraints of identification-based practices, some field scholars started to engage in generating social constructionist and nonnormative discourses of giftedness. By decoupling giftedness from the either-or reductionist psychometric model that simplifies giftedness as a static condition, emerging thoughts on giftedness see it: (a) as a socially constructed entity that constantly evolves with our society (i.e., different forms of giftedness emerge in different times and/or societies), (b) as an inclusive nonnormative guiding framework that seeks out each individual’s unique giftedness and talents, and (c) as a recursive person-in-situation realization that depends on the complexity of a system and the dynamism between an individual and his or her environment. This emerging nonreductionist view corresponds well to systemism—a worldview that sees everything as a system or a part of a system (cf. Banathy, 1992; Banathy & Jenlink, 2004; Bunge, 1996, 2000, 2004; Choi, 2011; B. Davis et al., 2015; Pickel, 2011; Wan, 2011). According to systemism, systems include features (e.g., interactions, relations, and mutual interdependencies) not possessed by their mechanical components. To put it simply, systemism embraces the Aristotelian notion of “the whole is greater than the sums of its parts.” While still adhering to a realist ontology (Choi, 2011; Wan, 2011), systemism holds a relational epistemology that intends to understand the world in a more-than-mechanical way. In accordance, the world is perceived as a network rather than a machine, metaphorically speaking (Capra & Luisi, 2014; Choi, 2011). In this new light of relational epistemology, giftedness is no longer approached as a self-contained psychometric entity/trait; rather, it is perceived as a fluid social construct that reflects the sociocultural values in a given time and context (a case in point is the current strong focus on the science, technology, engineering, and mathematics giftedness initiatives in North America). While giftedness is epistemized with systemic/relational thinking, the ideology of gifted education also switches from “education for the gifted” to “education that is gifted.”

Barab and Plucker (2002) rendered a convincing account of gifted education based on ecological psychology (see Barab & Roth, 2006; Gibson, 1977, 1986; Keating, 2009; Lave & Wenger, 1991)—a branch of psychology that accentuates systemic thinking. Barab and Plucker abandoned the normative notion of giftedness and espoused a person-in-situation epistemological framework that regards giftedness as a product of functional relations between a student and the environment in which he or she is situated. To accentuate the interactive nature of the realizations of possible functional relations, they adopted a relational terminology, such as environmental affordances and individual effectivities. According to Gibson (as cited in Barab and Plucker, 2002), an affordance is “a specific combination of properties of an environment, taken with reference to an individual, that can be acted upon” (p. 169), and reciprocally, an effectivity is “a specific combination of properties assembled by an individual, taken with reference to the environment, that allows for the dynamic actualization of a possibility for action” (Barab & Plucker, 2002, p. 169). In light of this “situated view” (Plucker & Callahan, 2014, p. 392), giftedness and talents are no longer perceived as static qualities awaiting identification and interventions. Instead, giftedness and talents are perceived as socioculturally constructed values, and the aim of education is to create various affordances, whether concrete or abstract, that help meet students’ effectivity profiles. For example, a student with an effectivity profile of strong logical–mathematical dispositions would benefit from the current educational milieu in North America that focuses strongly on science, technology, engineering, and mathematics education (Kettler, 2016). Every society endorses an affordance network that may or may not recognize a student’s effectivity set (one can simply imagine what would have happened if Mozart had not been born in a society that valued the importance of music).

Hymer (2012) proposed the GRACE model of gift creation (a reincarnated version of Hymer’s G-T CReATe model proposed in 2009). While Hymer (2009, 2012) and his colleagues (Hymer et al., 2009) did not generate an account of ontological discourse, the GRACE model of gift creation highlights the complex and dynamic process of gift transaction in a given social context and sees giftedness as a shared nonnormative quality among pupils. To capture the essence of gift transaction, Hymer composed his GRACE model with five verb form imperatives: Grow, Relate, Act, Challenge, and Exert. The model adopts a growth-focused and incremental approach to intelligence and argues that optimal growth happens when one or more of the following factors are present: good relational rapport, active participation in learning, dialectical/contradictory moments that present challenges, and a lasting motivation and persistence to exert one’s best.

The development of the gift transaction paradigm is in an embryonic stage. Essential elements that constitute the paradigm, such as definitions and curricular options, are still emerging and not easily found in the gifted literature. In terms of definitions, Eyre (2011) adopted a goal orientation point of view and sees giftedness as an end point rather than a starting point. More specifically, Barab and Plucker (2002) characterized giftedness and talents as “a set of functional relations distributed across person and context, and through which the person-in-situation appears knowledgably skillful” (p. 174). In other words, giftedness and talents are emphasized as “the *dynamic transactions* among the individual, the physical environment, and the sociocultural context” (Barab & Plucker, 2002, p. 174, italics added). Giftedness and talents are viewed as an optimal interactualized transaction between an individual and his or her environment—a dynamic proposition that distinctly departs from an identification-based dichotomous proposition (M. R. Coleman, 2013). In short, the essential undertaking of the gift transaction paradigm is to create a learning context that can afford students opportunities to transact their effectivity sets into giftedness.

In terms of services and provisions, two general directions that speak to the situated view of giftedness have been proposed. First, the importance of a rich context that will address and afford the multitudes of talents and abilities that come with the diverse body of students has been highlighted. For example, Barab and Plucker (2002) proposed that educators should “exercise the environment so that it contains numerous opportunities for action for an individual with the requisite abilities to act on these opportunities” (p. 167). Focusing on promoting the occurrence of functional transactions between an individual’s effectivities and the affordances of an environment, Barab and Roth (2006) emphasized the importance of designing a curriculum-based ecosystem wherein rich contextual specifics are provided to engage learning and life–world relevance (i.e., real-world problem solving) is embedded to inspire future planning. Interestingly, Barab and Plucker (2002) resituated Renzulli’s Schoolwide Enrichment Model (see Renzulli, 1977; Renzulli & Reis, 1997), a gifted education model developed in the 1970s during the identification paradigm, and demonstrated its relevance in the new paradigm by arguing that gifted education pedagogy can bring about individuality and uniqueness in all students through providing rich and broad learning experiences that enhance and create talents, a strong feature of the Schoolwide Enrichment Model.

Second, field curriculum developers are proposing curricula that address the concept of conditionality of giftedness by offering appropriately differentiated instruction for students who have advanced learning needs. For example, Peters and Matthews (2016) avoided the term gifted and adopted “advanced academics” (p. 55) instead to highlight the mismatch between learning needs and local district curriculum. In the advanced academics model, Peters and Matthews proposed the adoption of a Differentiation Educational Plan to address (locally relational) advanced learning needs regarding an academic subject/discipline, such as language arts, mathematics, science, and social studies. Similarly, VanTassel-Baska and Wood (2010; see VanTassel-Baska, 1986, for the foundation of this model) designed the Integrated Curriculum Model in which a student’s level of readiness for more advanced work is evaluated by a set of differential standards. This model implies that “ableness” is a topic- and time-bound state, rather than an ascribed prediction. Also focusing on differentiation strategies, Tomlinson et al. (2008, 2009) put forth the Parallel Curriculum Model that accentuates four interrelated yet parallel designs for organizing curriculum: Core (content understanding), Connections (interdisciplinary network), Practice (methods and skills), and Identity (content mastery as self-actualization). In Parallel Curriculum Model, all learners should have the opportunity to experience all these facets of knowledge and the curriculum should support a student’s developing expertise through ascending levels of intellectual demand. Moreover, some researchers in the field (e.g., Bell, Taylor, McCallum, Coles, & Hays, 2015; E. F. Brown & Abernathy, 2009; M. R. Coleman, 2013; M. R. Coleman & Hughes, 2009; Hughes & Rollins, 2009; Johnsen, 2011; McCallum et al., 2013; Pereles, Omdal, & Baldwin, 2009; Rollins, Mursky, Shan-Coltrane, & Johnsen, 2009) applied Response to Intervention (see Batsche et al., 2005), an emerging inclusive pedagogy, to cater to the learning needs of students who are more able.

As systemism urges us to perceive phenomena in terms of dynamic connectedness, relationships, patterns, and context, the construct of giftedness is gradually coming to be understood in this light. These emerging concerns of giftedness and gifted education speak to “interdependent conditionality” rather than “identifiable conditions.” Seeing giftedness as a complex system also poses challenges to educators in which they have to proactively create learning pathways by better understanding students’ effectivities and constructing an environment with sufficient affordances to recognize and nurture these effectivities. In light of this emergent thinking, the focus of gifted education has switched from “giftedness identification” to “giftedness transaction”—that is, recognizing the sociocultural nature of giftedness and focusing on creating an educational environment with affordances that effectuate the multitudes of giftedness and talents. In other words, gifted education is shifting gears from “education for the gifted” to “education that is gifted” to ensure every student is properly challenged.

## Closing Remarks

For changes to take place, we need to recognize how our taken-for-granted way of thinking from within the discipline’s meaning-making system impacts the educational process in perhaps unintended ways. (Gallagher, 1999, p. 69)

The field of gifted education is part of a larger system of global evolution in education; undergoing paradigm changes is natural (Cohen et al., 2000). This article provided a synthesized review of gifted education and research over the past century. We proposed a meta-theoretical paradigm shifts framework (PSF-GT) that consists of three distinct patterns of ideology, namely demystification, identification, and transaction. These patterns were explicated through historical events, educational and academic movements, and theories within and beyond the field. Through discerning these paradigm shifts, we noticed an escalating trend of inclusiveness (i.e., broadened notions of what giftedness is) and a gradual development of proactiveness (i.e., teachers become more involved with giftedness and talent development) in gifted education.

**Figure 2.**
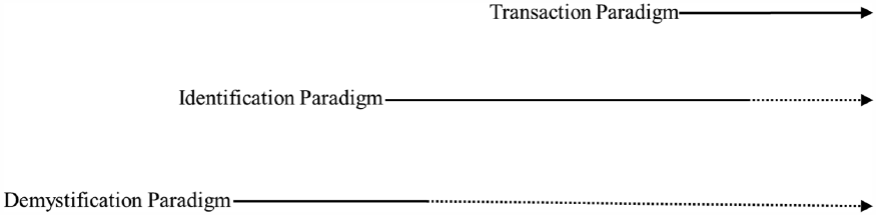
Overlaps and co-occurrences in the evolving paradigm shifts.

There are caveats to the article that should be considered before proceeding to the conclusion. First, while PSF-GT was constructed by examining changes in research, policies, and practices vis-à-vis shifts that have occurred in education and academia, it is not suggested that these are the only shifts that merit consideration. Second, although the paradigm shifts are presented as distinct stages that mirror chronological development, we by no means suggest that these shifts occur in abrupt and sudden ways. Rather, there are overlaps and co-occurrences (as illustrated in Figure 2) as no new paradigm occurs in a vacuum. A new paradigm is always based on the reflections on and critiques of a previous paradigm (or paradigms). We see paradigm shifts as a healthy evolution in our field in which they have broadened our horizons in understanding what giftedness is and/or what makes giftedness. In fact, many of us might adopt a mixture of different ideologies in our practices, knowingly or unknowingly. Third, it is important to note that we recognize the value and justification of each ideology in light of its historical context because each is a product of place and time. Last but not least, it is not our intent to propose PSF-GT as “the way” to see paradigm shifts in the field. Instead, the article renders an alternative approach for examining paradigm shifts in the field that adds to those put forth by many other field scholars (e.g., Cohen et al., 2000; Dai & Chen, 2013, 2014; Matthews & Dai, 2014; Matthews & Foster, 2006; Tannenbaum, 2000; Ziegler & Phillipson, 2012; Ziegler, Stoeger, & Vialle, 2012).

As Matthews and Foster (2006) noted, “A strong foundation gives us the solid footing that we need in order to define and pursue meaningful goals, and connect with revitalizing visions of possibility” (p. 64). We believe that PSF-GT can provide big-picture guidance for coordinating meaningful research and practice. That is, through a better understanding of the nature of paradigm shifts in the field, practitioners and policy makers will be able to (a) discern and justify the ways in which their work fits into broader educational and societal contexts and (b) generate meaningful goals and productive actions which are philosophically and theoretically grounded. We also hope this article stands as an opportunity for researchers to reflect on the progress that we have made in the field and suggest new research directions that address current issues and challenges. All in all, we see our work on paradigm shifts as part of an ongoing “philosophical meta-discourse” (Cohen et al., 2000, p. 334) that inspires productive actions and helps the field further clarify the scope and scale of gifted education.

## Notes

1. For example, *Creativity Research Journal, Exceptionality, Gifted Child Quarterly, Gifted Educational International, High Ability Studies, Journal for the Education of the Gifted*, and *Roeper Review*, to name a few.

2. Systemism in this article speaks to “soft” systems theories qua philosophy of (social) science and functions as an analytical paradigm or a set of heuristics. On the other hand, “hard” systems theories refer to substantive explanatory theories predominantly mathematically based and technology-oriented, such as cybernetics (theory of control systems), chaos theory, and game theory (see Pickel, 2011).

3. David Hume was among the first British empiricists. He argued that humans can have knowledge only of the objects of experience and the relations of ideas (see Hume, 1739/2000).

4. Auguste Comte is regarded as the founder of sociology and the most important popularizer of positive philosophy (Crotty, 1998/2011; Delanty & Strydom, 2003; Turner, 2003).

5. Both Plato in Greece and Confucius in China referred to exceptionalities as heavenly qualities (Ziegler & Heller, 2000).

6. Stern determined mental age by test performance, that is, a 7-year-old who performed like a 9-year-old was said to have a “mental age” of 9. Stern divided mental age by chronological age and multiplied the result by 100 to achieve the IQ.
